# Development of a Case-Based Learning Framework for Medical Education: A Scoping Review

**DOI:** 10.1007/s40670-025-02583-6

**Published:** 2026-01-15

**Authors:** Skye Nandi Adams, Kelly Ann Kater, Arshima Khan, Jenna Sher

**Affiliations:** Department of Speech-Language Pathology and Audiology, School of Human and Community Development, Faculty of Humanities, University of the Witwatersrand, Johannesburg, South Africa

**Keywords:** Case-based learning, Framework, Scoping review

## Abstract

Case-based learning (CBL) is widely used educational strategy in medical education for assessing students’ knowledge and clinical skills. Despite its benefits, there is an ongoing debate regarding best practices for developing cases. This study aims to synthesize current literature on CBL to develop a framework to support the creation of cases for theoretical and clinical courses. A scoping review, guided by the Arksey and O’Malley framework was conducted. Searches were conducted across five databases (CINAHL, ERIC, Scopus, and Google Scholar), and studies were included if they focused on CBL in health professions education. Thirty-five articles were in included in the final review. The included studies covered a range of disciplines, including medicine, nursing, physiotherapy, speech-language pathology, and interprofessional education. Studies were primarily from high-income countries with limited representation from low- and middle-income countries. A framework for CBL was developed encompassing five core components: content, structure, attributes and strategies, process and strategies, and outcomes. The proposed model facilitates student engagement, fosters reflective practice, and incorporates technological innovation to enhance learning. This framework offers practical guidance for educators in medical education seeking to design, adapt, and implement CBL approaches that are aligned with competency-based medical education. Future research may want to explore the application of CBL in the development of video simulated cases.

**Supplementary Information:**

The online version contains supplementary material available at 10.1007/s40670-025-02583-6.

## Introduction

Case-based learning (CBL) is a widely used educational strategy used in medical education to bridge theoretical knowledge with real-world clinical application. CBL fosters the development of students’ knowledge, reasoning, and decision-making skills [1]. Compared to conventional lecture-based teaching, CBL more effectively promotes critical thinking and problem-solving skills [2–5]. Well-constructed cases are authentic, aligned with course outcomes, and stimulate both reflection and discussion [6]. They reflect real-world complexity, incorporate diverse perspectives, and allow students to apply knowledge into practice. Traditionally, cases have been adapted from clinical encounters to ensure contextual relevance and accuracy, thereby enabling students to analyse patients, make inferences, and make decisions in situations that mirror professional practice [5, 7, 8]. Educational frameworks can support consistency and clarity in case development by outlining key components such as learning objectives, pedagogical strategies, feedback structures, and modalities of delivery. Such tools are especially valuable in competency-based education models, where alignment with assessment and clinical performance standards is essential. In addition, the use of frameworks can enhance both learner engagement and educational effectiveness. However, significant debate persists regarding the optimal development of cases and the necessity of facilitation for achieving intended educational outcomes.

Despite its pedagogical advantages, challenges persist in how CBL is designed and implemented. In some instances, the development of cases relies heavily on individual educator expertise and clinical experience, often in the absence structured guidance or pedagogical tools [9]. This has led to inconsistencies in case quality, depth, and alignment with curricular goals, particularly for early career academics and novice clinical educators [9]. In particular, the lack of standardisation in case structure, presentation style, and feedback mechanisms limits the potential of CBL to be scalable and integrated into the curriculum. Moreover, there is a call for CBL approaches to reflect the socio-cultural and systemic realities of diverse healthcare settings. Yet, many existing cases are not designed with cultural or contextual responsiveness in mind. As such, students may encounter difficulty relating to case content or applying learning to their own clinical environments, especially in low- and middle-income contexts. To address these gaps, recent literature has increasingly advocated for the use of structured frameworks in guiding case design [10, 11].

A frequently cited limitation in the literature regarding CBL is the additional time and effort required to prepare high-quality case materials [12–15]. By using a standardised CBL framework, educators can streamline the development process and ensure that cases follow a consistent, pedagogically sound structure. This promotes comparability, clarity, and coherence across teaching materials and enhances the interpretability of clinical cases for both students and educators [16, 17].

As lecturers in the department of speech language pathology at a South African university, we recognise the evolving educational landscape. Effective teaching must extend beyond the transfer of knowledge to prepare students for the realities of clinical practice in diverse clinical setting. Therefore, our decision to develop a culturally and contextually responsive CBL framework is informed by our first-hand experiences of the challenges involved in delivering relevant, engaging case material within both classroom and clinical settings [18].

Therefore, the aim of this research was twofold (1) to synthesize the literature on CBL in medical education and (2) to develop a framework for the creation of cases that can be applied to both theoretical and clinical courses. While this study was grounded in the context of speech-language pathology education, the framework is designed to be broadly applicable across allied health and medical education fields.

## Methodology

A scoping review was conducted to explore the existing literature on the development of written cases in medical education, with the aim of constructing a comprehensive framework that can be used for crafting cases. This methodology was chosen due to its suitability for exploring complex and contextually embedded educational phenomena. The review followed the methodological framework outlined by Arksey and O’Malley [19] as updated by Levac et al., [20] encompassing five stages: (1) identifying the research question, (2) identifying relevant studies, (3) study selection, (4) charting the data, (5) collating, summarising, and reporting the results used to conduct the scoping review, and (6) consultation. An ethics waiver was granted by the University XXX Human Research Ethics Committee (HRECNMW24/06/03).

### Identifying the Research Question

The research questions for this scoping review were determined using the Population–Concept–Context (PCC) framework, as recommended by the Joanna Briggs Institute (JBI) for guiding scoping reviews. This included (1) Population (P): Clinical educators and academics involved in medical and health professions education, (2) Concept (C): Development and use of case-based learning, including frameworks, models, and strategies for case design, (3) Context (C): Theoretical and clinical teaching within medical and rehabilitation education settings globally. Two guiding research questions were established: (1) What evidence exists in the literature regarding strategies, models, or frameworks for the development of case-based learning? (2) How can a culturally and contextually responsive framework be developed to guide the creation of cases?

### Identifying Relevant Studies

The studies that were included in this review needed to meet the following inclusion criteria: (1) addressed CBL or frameworks related to CBL; (2) were situated in the field of education and rehabilitation sciences (including medicine and nursing); (3) were published in English, (4) fell within the time-period between 2014–2024, (5) were peer reviewed journal articles using primary study designs (qualitative, quantitative, or mixed-methods) were included. Grey literature and non-academic sources were excluded to ensure quality and reproducibility.

A systematic electronic search for articles was conducted on 23–24 May 2024 (JS & KK). Three databases were used: Cumulative Index to Nursing and Allied Health Literature (CINAHL) allied and nursing, Scopus, ERIC, as well as the first five pages of google scholar. The databases were selected based on their relevance to education and health sciences. The search strategy incorporated Boolean operators (AND/OR), truncation symbols (e.g., * to retrieve multiple word endings), and proximity operators where applicable, tailored to each database’s syntax and functionality. For example, terms such as "case-based learning" OR “clinical case” were combined with “framework” and discipline-specific terms like “healthcare education” OR “medical education”. Subject headings were further refined to optimise the search results for each database. This ensured both keyword and concept-based retrieval. An example of the search strategy used in CINAHL is detailed in Table 1. To ensure methodological rigour and optimise the sensitivity and specificity of the search, the initial draft of the search strategy was reviewed by an experienced academic librarian with expertise in health sciences and systematic searching. Following the review, no changes were deemed necessary, as the search terms were already appropriately tailored to each database. As a result, the original strategy was retained.

**Table 1:** Keywords for database searches

	Research database terms
Key concept (1)	“Case Based Learning” OR “Clinical Case” OR “Patient Scenario” OR “Clinical Scenario”
Key concept (2)	“Framework” OR “Model” OR “Methodology” OR “Approach”
Key concept (3)	“Medical Education” OR “Clinical Education” OR “Healthcare Education”
Context	Global context

### Study Selection

Following the database searches, all identified peer-reviewed results were collated and uploaded into Mendeley. The initial search results yielded the following results: CINAHL allied and nursing (*n* = 611), Scopus (*n* = 338), ERIC (*n* = 345), and google scholar (*n* = 29). Supplementary searches were conducted on Google Scholar using a simplified version or multiple simplified versions of the search string and screening of the first five pages. Additionally, citation tracing was conducted which included a review of references of studies included after full text screening. After initial screening 1323 articles were identified. Duplicates (*n* = 510) were removed leaving 813 articles to screen. Titles and abstracts were screened by two independent reviewers (KK, SA), with an additional reviewer providing an independent secondary review for assessment against the inclusion/exclusion criteria (AK). Peer-reviewed results that did not meet the inclusion criteria were excluded. Articles were excluded if they did not relate to CBL development, focus primarily on the impact of case-based learning*,* or focused more on the learning outcomes and student perceptions rather than on developing or discussing cases for CBL. In addition, articles were excluded if they discussed a case study methodology, did not focus on student training in higher education, and were in different disciplines.

Any disagreements that arose between the reviewers were resolved through discussion and input from an independent reviewer until consensus was achieved. Following titles and abstract screening an additional 724 articles were excluded and 89 articles were included in the full text review. Reviews and book chapters were read to identify additional primary articles, but were excluded from final analysis. Following full text review a further 54 articles were excluded and 35 articles were included for the final review. An iterative approach to study screening and selection was employed to emphasise a more inclusive final list of studies*.* All articles were accessed electronically. Consistent with the methodological framework for scoping reviews proposed by Arksey and O’Malley and further developed by Levac et al. and the Joanna Briggs Institute (JBI), no formal critical appraisal of study quality was conducted [19]. The decision to not include a formal appraisal aligns with the exploratory and inclusive nature of the review, which sought to capture a wide range of perspectives and approaches to case-based learning.

**Fig. 1:**
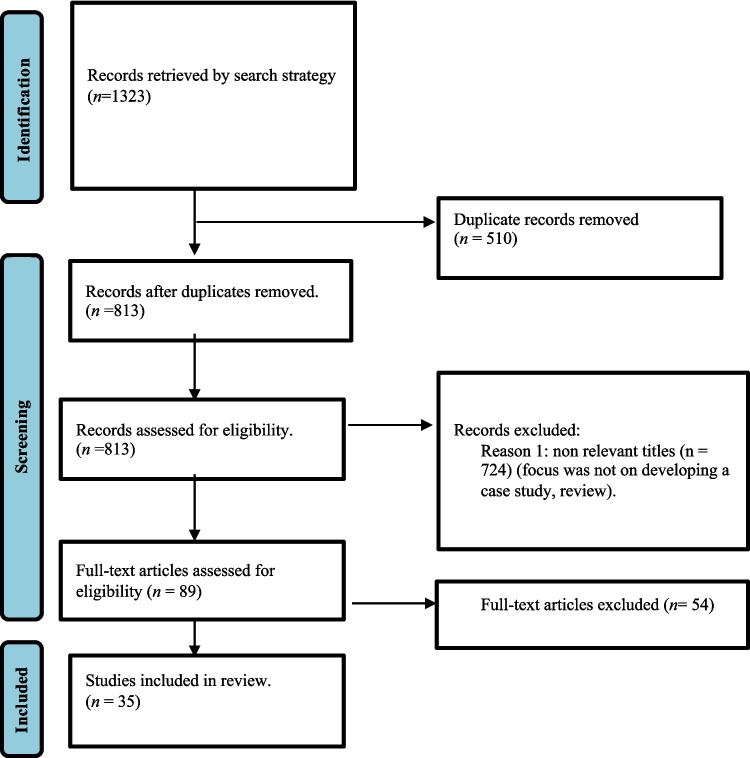
Preferred reporting items for systematic reviews and meta-analysis flow diagram for scoping reviews (PRISMA-ScR)

The Preferred Reporting Items for Systematic reviews and Meta-Analyses extension for Scoping Reviews (PRISMA-ScR) diagram in Fig. 1 outlines the process that led to the selection of 35 sources of evidence for inclusion in the review.

### Charting the Data

All authors contributed to designing the data charting template. The data extracted included specific details about the study objectives. Article title, abstract, full text were screened independently by two researchers according to the inclusion and exclusion criteria, respectively, and inclusion or exclusion of studies was decided jointly by all members of this study participating in discussion. Data were extracted according to a uniform data extraction form including: author and date, context, methodology, and discipline being studied.

Building on the work of Kim et al. [5] who developed a conceptual framework for the teaching of case development across disciplines, this study extends that model by synthesizing more recent literature and focusing specifically on student training within the fields of medicine and rehabilitation in higher education contexts. Data was extracted under five key components: (1) content, (2) structure, (3) process and attributes, (4) process and strategies, and (5) outcomes.

Independent categorisation was conducted by two reviewers (KK, SA) based on the study objectives and previous research. A content analysis was conducted using the framework by Elo & Kyngäs [21]. Codes were drawn from both inductive and deductive coding informed by the literature. Each framework component was defined according to the literature (content, structure, attribute, process, and outcome) [3, 5]. Ten articles were included in the initial codebook and each article was coded and categorised according to each individual component by each author individually. An example of this was under content, first the authors defined ‘content’ and then added codes that were relevant to that component such as student instruction, learning outcomes, patient information, and assessment details. Each of these were defined and included in the codebook (Table 2). We then checked all the codes from both authors. The same two researchers compared their codes and cross-checked their work to ensure no repetition of codes in the different codes before finalising the grouping under each category. To ensure coding reliability, two independent reviewers screened and extracted data from a subset of studies (*n* = 15) using a predefined extraction form. Initial agreement was calculated, with percentage agreement reaching 90%. To provide a more robust measure that accounts for chance agreement, Cohen’s kappa was calculated, resulting in a value of κ = 0.82, which indicates substantial agreement. Discrepancies were discussed and resolved through consensus, and coding was refined accordingly before continuing with the remaining articles. As a scoping review methodology is iterative, this allowed for an adjustment to be made regarding the inclusion of relevant studies during consensus discussions [20].

**Table 2:**
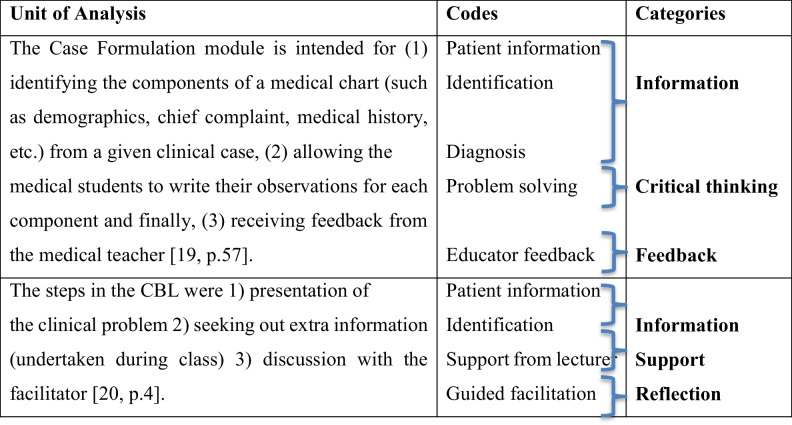
Example of the analytical process

Each article was then analysed independently by each reviewer using the codes that were developed (see Supplementary Material 1). A copy of the codebook showing the codes, categories, and frequency of each code can be found in Supplementary Material 1.Where a specific code was mentioned in the article, this was highlighted and a tally was done to understand the frequency of each code. Beyond frequency counts, we considered thematic salience, where codes were prioritized not just by how often they occurred but by their conceptual contribution to the development of the framework. Thematic saturation was deemed achieved when no new codes or categories emerged from the analysis of the final third of the included studies. Codes that were identified across a range of disciplines, geographical regions, or CBL formats were considered conceptually robust and relevant. This approach ensured that the final framework captured both widely adopted practices and those with strong theoretical or instructional value. All five components of the framework were retained based on their consistency across the literature, while individual codes within these components were selected based on a combination of frequency, saturation, and depth of conceptual insight.

## Results

### Study Characteristics

The 35 included studies were published between 2014 and 2024 (Table 3). The highest number of studies were published in 2021 (*n* = 5) and 2022 (*n* = 6). Publications originated from a broad range of countries, including Australia, Belgium, Canada, China, Croatia, India, Iran, Italy, Korea, Lebanon, Mexico, the United Kingdom, the United States, and Austria. The United States (*n* = 10) and China (*n* = 8) had the highest number of publications. Where multiple author affiliations or multinational contexts were present, both countries were recorded.

**Table 3:** Characteristics of included studies (*N* = 35)

No	Author	Year	Country	Study design	Discipline
1	Adams et al.,	2014	Australia	Mixed -methods	Physiotherapy
2	Braeckman et al.,	2014	Belgium	Quantitative	Occupational medicine
3	Yoo & Park	2014	Korea	Quantitative	Nursing
4	Goldberg et al.,	2015	Australia	Descriptive	Healthcare
5	Kantar & Massouh	2015	Lebanon	Qualitative	Nursing
6	Kukolja Taradi & Taradi	2015	Croatia	Quantitative	Physiology
7	Imai et al.,	2016	Canada	Qualitative	Dental Sciences
8	Ricci et al.,	2016	Italy	Descriptive	Healthcare
9	Trommelen et al.,	2017	United States	Quantitative	Physical Therapy
10	Ali et al	2018	Australia	Quantitative	Medical Education
11	Jauregul et al.,	2018	United States	Quantitative	Medicine
12	Pilcher	2018	United States	Qualitative	Nursing
13	Ridley & Byrom	2018	United Kingdom	Descriptive	Midwifery
14	Kumar et al.,	2019	India	Qualitative	Medicine
15	Li et al.,	2019	China	Quantitative	Nursing
16	Turk et al.,	2019	Austria	Quantitative	Medicine
17	Wadowski et al.,	2019	Austria	Descriptive	Psychiatry
18	Grover et al.,	2020	India	Quantitative	Pathology
19	Şen Akbulut & Hill	2020	United States	Qualitative	Education
20	Zhu et al.,	2020	China	Quantitative	Nursing
21	Bavaria, De Dhaem & Shaefer	2021	United States	Observational	Dentistry
22	Burgess et al	2021	Australia	Mixed-methods	Medicine
23	Diel et al.,	2021	United States	Quantitative	Ophthalmology
24	Gholami et al.,	2021	Iran	Quantitative	Nursing
25	Major et al	2021	United States	Quantitative	Medical Education
26	Koehler et al.,	2022	United States, China	Case study	Education
27	Ma & Zhou	2022	China	Quantitative	Nursing
28	Okoro et al.,	2022	United States	Qualitative	Pharmacy
29	Perez et al.,	2022	Canada	Qualitative	Dentistry
30	Yao et al.,	2022	China	Qualitative	Nursing
31	Harden & Pronchnow	2023	United States	Descriptive	Nursing
32	Yao et al.,	2023	China	Qualitative	Nursing
33	Aguayo et al.,	2024	Mexico	Qualitative	Dentistry
34	Xu et al.,	2024	China	Mixed-methods	Nursing
35	Zhao et al.,	2024	China	Quantitative	Midwifery

US-based studies demonstrated a balanced emphasis across all framework components, but particularly elevated use of simulation-based content and interactive learning strategies. Whereas, LMIC-based studies (Lebanon, India, Iran) often highlighted context-sensitive challenges and adaptable formats, supporting the inclusion of cultural responsiveness as a core principle.

A summary of study characteristics are presented in Table 4. Studies came from different disciplines with nursing (*n* = 11, 31.4%), medicine and medical education (*n* = 5, 14.3%) being the most frequently studied disciplines highlighting the focus in CBL studies for the field of medical education. Additionally, studies used a variety of study methodologies with the most commonly being including quantitative (*n* = 15, 43%) and qualitative (*n* = 10, 29%). Mixed-methods and qualitative designs, especially in nursing, often yielded rich narrative detail on implementation strategies and learner experiences, informing our coding on *attributes and learning strategies* and *support strategies*. In contrast, quantitative studies from medicine focused on case structure and learning outcomes, influencing *conten*t and *outcomes* used to support to support clinical reasoning development.

**Table 4:** Summary of characteristics of included studies (*N* = 35)

Characteristics	*N*	%
Study Discipline		
Nursing	11	31
Medicine	4	11
Dentistry	3	9
Healthcare	2	6
Medical Education	2	6
Midwifery	2	6
Education	2	6
Physiotherapy	1	3
Occupational Medicine	1	3
Physiology	1	3
Dental Sciences	1	3
Physical Therapy	1	3
Psychiatry	1	3
Pathology	1	3
Ophthalmology	1	3
Pharmacy	1	3
Study Design		
Qualitative	10	29
Quantitative	15	43
Mixed methods	3	9
Descriptive	5	14
Observational	1	3
Case study	1	3

### Development of a Case Based Teaching Framework

This framework presents the primary findings of a scoping review that synthesized best practices and pedagogical principles from the literature on CBL. This framework builds on from existing frameworks [5, 22–24] as it specifically targets health professions education and reflects competencies and pedagogical practices unique to clinical and rehabilitation education. This framework has been designed to guide educators *step-by-step* in the development, implementation, and evaluation of effective and contextually relevant case studies in medical and rehabilitation education. The framework is organized around five key components: (1) content, (2) structure and presentation (3) attributes and learning strategies, (4) support strategies, and (5) outcomes. For each component, a definition is provided and a summary of actionable guidance with rationale from the review.

**Component 1: Content**

Content was defined as information that should be included in the case. Content considerations included the development of the case, information to be included, and contextual relevance.

1. **Decide the use of real vs developed case**: Choose between a real clinical case or one that is developed based on what is being observed at the clinical site. Decide on how many perspectives to include and how. Cases should be developed in order to match the clinical and/or theoretical outcomes [10, 19–21, 25–38]. The case can be developed or based on a real case and include real patient information [20, 25, 28, 32, 39]
2. **Essential information:** Decide what information to include in the case. It is imperative to tailor the case according to the learners experience (e.g., first-year vs. final-year students) and to match it to lecturers course outcomes regarding the level of detail that might be required. Information to include:
  Learning Outcomes [25, 28, 40, 41]
  Student instruction [28, 40]
  Patient information [21, 29, 42]
  Diagnostic including differential diagnoses [25, 27, 29, 33, 42]
  Assessment details [10, 25, 29, 43]
  Management information including possible outcomes if available [25, 27, 29, 33, 39, 42–44].
3. **Contextual relevance:** Reflect local health issues, socioeconomic factors, and resource constraints (e.g., HIV/AIDS, TB, or access to healthcare) [43–46]. Ensure diverse cultural, linguistic and socioeconomic realities are representative of what students are likely to encounter in their healthcare settings.

**Component 2: Structure and Presentation**

Structure was defined as *how* the case should be delivered to students. Considerations include the format, sequencing and modalities used to convey case content and to encourage active engagement with the case:

1. **Select the case format:** Choose between an online case [10, 20, 28, 30, 45], written/paper based case [21, 41, 44], or collective case [19, 39, 40]. When choosing a case it is important to consider the alignment to course outcomes and availability of resources. In addition, it helps to include different modalities to better support student learning.
2. **Choose a presentation style:** Decide between an unfolding/progressive case [31, 33, 47] or a case with redacted information [26]. Using unfolding techniques, where new information is provided as the case progresses, mimic real-life patient encounters and can simulate local health systems, referral pathways and different healthcare contexts [45]. Redacted information cases trigger student enquiry. Consider incorporating multiple perspectives through the use of role playing [36, 41], real patients [48], and/or simulated patients [26, 28, 29].
3. **Use multimedia and digital tools:** Consider the use of multimedia and digital tools such as online case-based discussions or simulations. Using multimedia can enhance learning by providing students with additional time to engage critically with the material and reflect on their clinical reasoning processes. Importantly, these tools also offer opportunities to embed culturally and contextually relevant content such as, region-specific health issues, diverse patient identities, and language variations into the learning experience [48].

**Component 3: Attributes and Learning Strategies**

Attributes and strategies refers to the pedagogical characteristics and cognitive scaffolding embedded in the case to facilitate learning and skill development. Component 3 focuses on cases not only being content-rich but also pedagogically robust, fostering development of deeper learning and transferable clinical skills.

1. **Promote critical thinking:** Use open-ended prompts that require reasoning and do not rely on recall. The incorporation of critical thinking is essential for developing competent, reflective, and adaptive healthcare professionals [19–21, 25, 26, 32, 39, 40]. Critical thinking fosters higher order thinking, clinical reasoning, problem solving, meaningful learning, and developmental reasoning and preparation [39]. By embedding critical thinking in CBL, educators can ensure that students are not just memorising information but applying knowledge and integrating theoretical content to clinical practice.
2. **Encourage self- reflection:** Add reflection through the use of self-evaluations, guided self-reflections, journals or debriefings. The inclusion of self-reflection is an important part of using cases as it promotes continuous professional growth, emotional intelligence, resilience, and ethical integrity [25, 30, 49]. It allows individuals to critically assess their decisions, refine their skills, and adapt to new knowledge.
3. **Align to learning taxonomies:** Include structured ways to analyse, interpret, and apply knowledge through the integration of theories to support learning [11]. The current framework uses two developmental theories (1) Bloom’s taxonomy [50] and (2) Millers pyramid [51]. The integration of theories ensures that students engage deeply with content, think critically, and develop problem-solving skills. In the framework the authors have integrated Millers pyramid to show how elements progress from basic knowledge to practical application. Miller’s original pyramid (Knows → Knows How → Shows How → Does) is a widely used framework for assessing clinical competence [50, 52]. Blooms taxonomy has also been included to show the different levels of learning from remember (low level) to create (high level).

**Component 4: Support Strategies**

Support strategies refer to the structured approaches, methods, and techniques used to facilitate CBL in practice. Support strategies create an effective framework for structured learning and continuous learning and improvement.

1. **Develop feedback mechanisms:** Design feedback mechanisms through peer feedback, educator feedback, and self-reflective feedback. Feedback can be incorporated through small group discussions, structured discussions, as well as guided and facilitated discussions [10, 26, 27, 39, 40, 42, 45, 49, 53]. Feedback should be considered based on the level of interaction and resources available. Feedback is a crucial element in the development of CBL and allows students to refine their knowledge, skills, improve their confidence, competency and learning [10, 31, 40, 42, 47, 48].
2. **Integrate support systems:** Use support mechanisms such as mentorship, collaboration, online support, peer support, and support from a lecturer or supervisor. Different support measures should be put in place as intentional methods to enhance learning, critical thinking, and performance [27, 33].

**Component 5: Outcomes**

Outcomes refer to the knowledge, skills and competencies that students develop through CBL.

4. **Define direct outcomes:** Include measurable outcomes such as quizzes or oral examinations. It is beneficial to include some measurable outcomes in the development of the case. Direct outcomes are those that can be assessed. Direct outcomes include improved knowledge, skill, competency, preparation, improved knowledge transfer to clinical setting and better student engagement [19, 25, 29, 31, 34, 39, 40, 42, 45].
5. **Identify indirect outcomes:** Indirect outcomes are those that are not directly measurable but may be secondary or in response to direct outcomes. It is important to observe indirect outcomes such as improved student confidence, improved integration of theoretical and clinical knowledge, improved communication skills, reduced anxiety, improved critical thinking, improved clinical reasoning, and improved motivation [10, 25, 27].

**Fig. 2:**
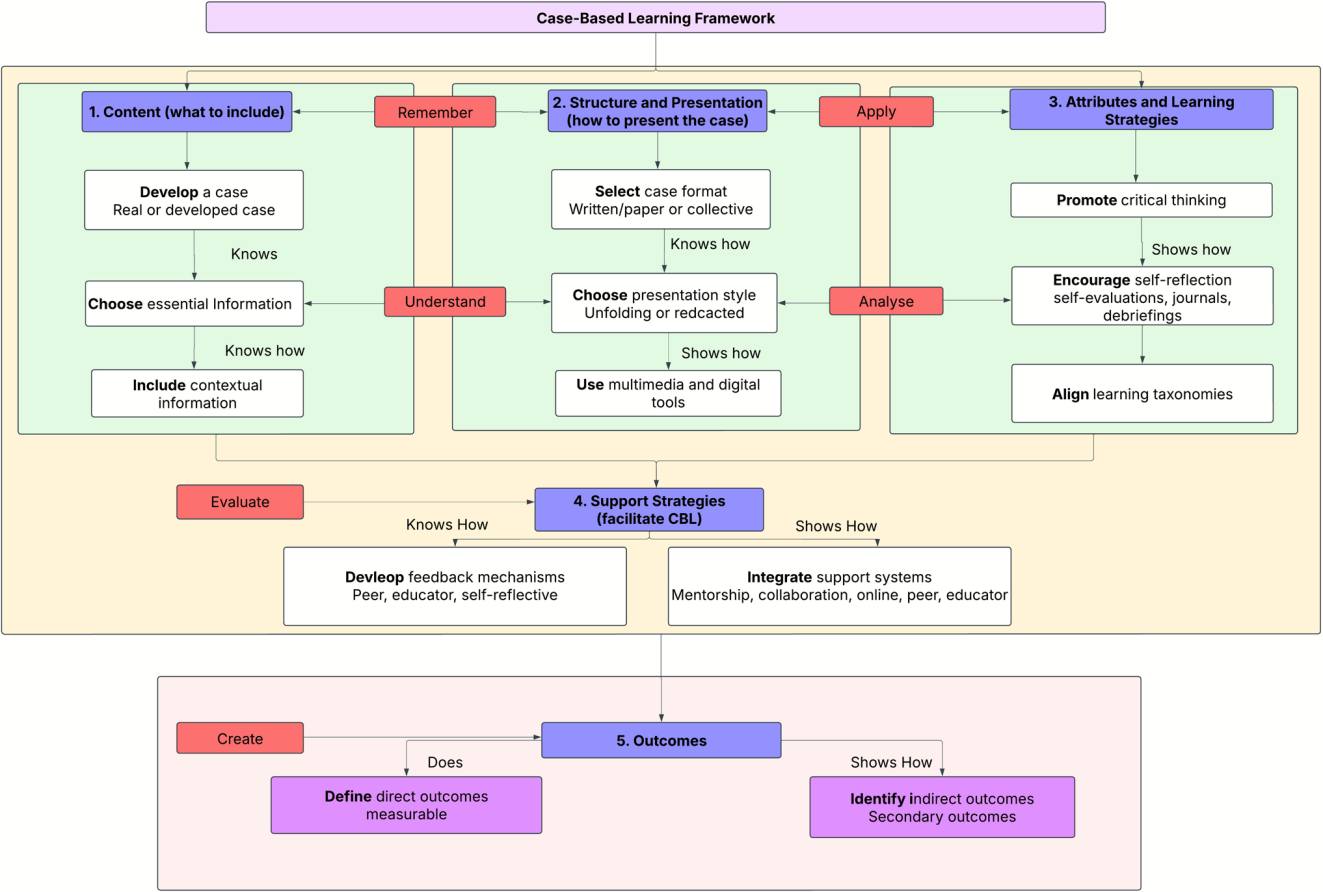
Conceptual framework of case based learning

Based on the findings, the five components were used to develop the framework presented in Fig. 2. The components were found to be interdependent and instrumental in designing effective, contextually relevant case.

### Consultation

In line with the enhanced scoping review framework by Levac et al. [20], we acknowledge the importance of stakeholder consultation as a sixth stage. The framework was piloted with eight subject matter experts; four clinical educators and four academic staff involved in speech language pathology education and clinical training. To develop a comprehensive framework that reflected current requirements and competencies in clinical education, the consultation phase was guided by the following research questions: (1) What components and pedagogical strategies are considered essential by educators and clinicians for effective CBL in medical and rehabilitation education?

To begin we shared a brief report with the subject matter experts outlining the results of the scoping review and the framework presented. Following this we had a meeting to review the findings and the different components identified in the literature. In order to assess the usability of the framework and integrate additional feedback, subject matter experts participated in the development of two cases—one paediatric and one adult—designed to reflect varied clinical settings and student needs. Subject matter experts were invited to provide structured feedback on its usability, relevance, clarity, and adaptability within their teaching contexts. This engagement added value by exploring the framework’s practical utility, uncovering context-specific considerations not previously captured in the literature, and enhancing its transferability across disciplines and clinical settings. Finally, a reflective focus group was held to get feedback from subject matter experts. Participants were asked to reflect on their experience co-developing case materials using the framework, using Driscoll’s reflective model ("What?", "So what?", "Now what?") as a guide [54].

The consultation revealed strong support for the structure, clarity, and adaptability of the framework. Participants noted that the framework allowed for a comprehensive and logical approach to case development that could be tailored to different levels of student training. Importantly, the framework was described as "*user-friendly*" and not overly prescriptive, enabling its application across varied modalities including simulations, lectures, and video-based learning. Educators highlighted the value of thinking holistically about a patient’s journey as reported by one participant, “*Using the framework was really interesting to actually think of it from the start and what this patient might go through in this journey… trying to kind of think about who this patient is, what would some of the things they are presenting with and how to then develop this into a case for students*” (Clinical Educator). Educators appreciated how the framework helped scaffold content based on learner needs and educational outcomes. One participant stated how the framework was used to develop a case and how it could be adapted for use with different student years or courses, “*We gave a lot of detail, but information can be added to it. So we can change it for a particular course or we can even remove some information for other student years – it allows us to scaffold*” (Academic and Clinical Educator). Despite this positive feedback, participants also raised the importance of validating the framework with student input. As such, future phases of the project will include structured student focus groups and surveys to further refine the framework.

## Discussion

The current research has been able to synthesize the available literature and provide a clear guideline on how to develop cases and integrate theoretical frameworks to support the use of CBL. Several key findings emerged out of this study. Most studies focused on nursing and medicine with a range of countries conducting research on CBL. This study highlighted the multiplicity in which CBL can be used and integrated into theoretical and clinical courses and the need to move away from didactic and traditional forms of teaching. Furthermore, the use of CBL allows the academic to facilitate learning rather than primarily teach, which is important for critical thinking, problem solving and engagement [8, 51, 55].

While previous frameworks [5, 22–24] have laid important groundwork by conceptualising the role of case development in higher education and targeting core skills such as critical thinking, the current framework builds on and significantly extends this work in several key ways. Firstly, unlike earlier models that primarily emphasized content alignment and instructional goals, our framework introduces a multidimensional structure that integrates content, structure, attributes and strategies, process, and outcomes, offering a more holistic and applied approach that is more appropriate in medical education and not just higher education. Secondly, it advances the field by explicitly incorporating technological integration, including the use of digital simulations and unfolding online cases, which are critical for contemporary health education settings, especially in resource-constrained or remote environments. Lastly, and perhaps most importantly, this framework foregrounds contextual relevance, drawing attention to socio-economic and cultural realities, such as limited access to healthcare, that are often overlooked in Western-centric models. Therefore, this framework responds to the need for case-based learning that are not only evidence-informed but also responsive to local contexts and structured to promote both competence and critical thinking.

Case-based learning remains an integral pedagogical strategy in health professions education, known for enhancing student engagement, clinical reasoning, and reflective practice [51, 54, 56] However, using CBL in practice can often be difficult to conceptualise and develop [57]. Therefore, the current study builds on prior conceptual models [5] on case development, by incorporating an updated review of the literature and integration of pedagogical approaches. The framework provided allows academics to integrate CBL into their theoretical and clinical teachings. In addition, the integration of theoretical frameworks allows academics to adjust, adapt, and tailor cases to suit the needs of their students depending on their year of study or complexity of the case. The integration of theoretical frameworks is critical in order to ensure students are engaging at the right level of critical thinking and to tailor CBL to the students ability and competencies being assessed. Bloom’s taxonomy describes cognitive learning progression, while Miller’s pyramid illustrates competency development in clinical practice. Integrating these frameworks enhances medical education by mapping cognitive skills to clinical competency, reinforcing the transition from theoretical understanding and exposure to competency based learning [13, 58].

A key contribution of this study is the emphasis on contextual relevance in the development of cases, particularly in addressing local healthcare challenges such as access disparities, socioeconomic constraints, and disease prevalence. Prior research highlights that context-specific cases improve student engagement and their ability to navigate real-world clinical complexities [43, 45, 46]. The framework is intended to guide both clinical educators and academic staff in the development of cases, including those based on real patients, in order to promote a patient-centered and practically grounded approach to CBL.

The incorporation of soft skills and reflective practice into the framework provides an advancement in case-based learning models. While traditional case-based approaches prioritise medical knowledge, integrating self-reflection fosters a deeper learning process, allowing students to critically evaluate their decision-making. This aligns with contemporary discussions on competency-based medical education, which advocate for holistic learning approaches [27, 35]. Research has shown that indirect outcomes are just as important as direct outcomes, particularly regarding students in medical education after graduation and into independent practice [56, 59]. Indirect outcomes impact student motivation, learning experiences, and cognitive learning outcomes [57]. Therefore, the framework should be used to enhance student learning and provide support to academics in using case-based learning approaches for both theory and clinical courses.

## Conclusion

This study presents an enhanced case based teaching framework that integrates content, structure, attributes, process strategies, and outcomes in a comprehensive manner. Research indicates that using a structured method can provide support to clinical educators that can improve teaching and learning experiences for themselves as well as students. The findings contribute to the ongoing evolution of medical and rehabilitation education by advocating for case-based learning models and frameworks that are student-centred, reflective, and technologically integrated.

This study is not without its limitations. Firstly, the scoping review methodology may have introduced selection bias. The review was limited to English-language publications and did not include grey literature, which may have excluded relevant studies from non-English-speaking contexts or those disseminated through alternative platforms such as institutional reports or conference proceedings. This may limit the global applicability of the findings, particularly in multilingual and low-resource settings. Secondly, the current study did not include any data or information on student perspectives. Input from students, would have added valuable insight into the practical relevance, usability, and contextual adaptability of the framework. Thirdly, the study focused on CBL in written formats and did not focus on other instructional modalities such as video-based learning or simulations, which are increasingly relevant in digitally enhanced health education. Although the framework allows for adaptation across modalities, empirical research is still needed to explore its effectiveness when applied to multimedia and immersive formats. Finally, the framework has not yet been empirically tested in educational settings. It remains a conceptual model that requires pilot implementation, integration into curriculum design, and the development of robust evaluation metrics to assess its effectiveness in enhancing learning outcomes.

Future studies may want to explore the application of CBL the development of video cases, in simulations, and in virtual reality in order to provide an innovative approach to experiential learning in the field of medical education. These formats offer immersive, experiential learning opportunities that mirror real-life clinical encounters and can be especially valuable for developing clinical reasoning, communication, and decision-making skills in safe, controlled environments. The use of simulation aligns with current trends in health professions education toward multimodal, learner-centered pedagogies. Future work should also include pilot testing of the framework across diverse educational contexts to assess feasibility, usability, and alignment with learning outcomes. This would allow for refinement of the framework based on practical implementation challenges. Furthermore, its integration into formal curriculum design should be systematically explored to ensure relevance across different levels of training (e.g., undergraduate vs postgraduate) and disciplines (e.g., speech therapy, medicine, nursing).

## Supplementary Information

Below is the link to the electronic supplementary material.

Supplementary file1 (DOCX 19 KB)

## Data Availability

The data that support the findings of this study are available from the corresponding author (S.N.A.), upon reasonable request.
