# Supplementary material for: Development of a Case-Based Learning Framework for Medical Education: A Scoping Review
Source: Med Sci Educ. 2026 Jan 15;36(2):975–87. doi: 10.1007/s40670-025-02583-6 (PMC13197500; doi:10.1007/s40670-025-02583-6)
Supplement: Supplementary file 1 — Supplementary file1 (DOCX 19 KB) [file 40670_2025_2583_MOESM1_ESM.docx]

**Supplementary Material 1. Codebook**

| **Content to be included** | **No.** | **Structure required** | **No.** | **Attribute and strategies** | **No.** | **Process and strategies** | **No.** | **Outcomes** | **No.** |
| --- | --- | --- | --- | --- | --- | --- | --- | --- | --- |
| Scenario development   1. Real case scenario 2. Developed case scenario | 13  27 | Type of case:   1. Online case-based studies 2. Collective case studies 3. Written/paper cases | 11  4  5 | Critical thinking:   1. Higher order thinking 2. Clinical reasoning 3. Problem solving 4. Meaningful learning 5. Developmental reasoning and preparation | 8  11  5  5 | Support:   1. Collaboration 2. Support from lecturer 3. Mentorship 4. Online support 5. Peer support | 8  4  3  2  3 | Direct Improvement:  1. Improved knowledge  2. Improved skill  4. Improved preparation  5. Knowledge transfer to clinical setting  6. Better student engagement  7. Improved self-directed learning | 9  10  1  2  2  1 |
| Information:  3. Student instructions  4. Learning outcomes  5. Patient information  6. Identification  7. Assessment  8. Management  9. Differential diagnosis | 2  5  3  7  4  8  5 | Case Presentation:   1. Unfolding/ progressive case studies 2. Redacted information | 3  3 | Self-reflection:   1. Self-evaluation 2. Guided facilitation 3. Student-centered | 2  2  3 | Feedback:  6. Peer feedback  7.Educator feedback  8. Reflective and self-evaluation | 2  8  9 | Indirect outcomes:   1. Improved student confidence 2. Improved integration of theoretical and clinical knowledge 3. Improved communication 4. Reduced anxiety 5. Improved critical thinking 6. Improved clinical reasoning 7. Improved motivation | 2  6  1  1  6  1  8 |
| Soft Skills:   1. Communication 2. Confidence | 2  3 | Length:   1. One-off session 2. Multiple sessions | 2  5 | Approach:  9. Developmental  10. Graded level of complexity (e.g. blooms)  11. Interdisciplinary | 6  1  3 |  |  |  |  |
| 11. Contextual information | 2 | Type of patients:  8. Real patients  9. Role playing   1. Simulated patients | 1  3  4 |  |  |  |  |  |  |
|  |  | Facilitated learning:   1. Structured discussion 2. Small group discussion 3. Facilitated discussion 4. Self-reflections | 2  11  4  6 |  |  |  |  |  |  |
